# A New Classification of Dermatochalasis, the Effect of This Classification and Blepharoplasty Surgery on the Visual Field

**DOI:** 10.5152/eurasianjmed.2025.25955

**Published:** 2025-10-13

**Authors:** Betül Dertsiz Kozan

**Affiliations:** Department of Ophtalmology, Gazi Yaşargil Training and Research Hospital, Diyarbakır, Türkiye

**Keywords:** Blepharoplasty, classification, dermatochalasis, visual field

## Abstract

**Background:**

The aim is to create a new classification according to the contact and effect of dermatochalasis on the eyelashes and to examine the effect of blepharoplasty surgery on the visual field (VF) in these stages.

**Methods:**

Demographic characteristics of cases that were staged as dermatochalasis (group 1) (stage 1), (group 2) (stage 2), and (group 3) (stage 3) between February 2025 and April 2025 were examined, and changes in the VF before and after blepharoplasty were evaluated. Stage 1 was accepted as dermatochalasis only; stage 2 as dermatochalasis with eyelash contact but no eyelash inversion; and stage 3 as dermatochalasis creating inversion in the eyelashes. The Humphrey 30-2 test was applied in the VF. Mean deviation (MD), pattern standard deviation (PSD), visual field index (VFI), and upper half VF sensitivity were evaluated. Those with refractive error and additional eye diseases were not included in the study.

**Results:**

A total of 60 cases were examined, 20 in each group. The mean age of the cases was 42.7 ± 14.9 (28-75). Forty of the cases were female and 20 were male. The mean age was higher in group 3, and there was a statistically significant difference (*P* < 0.05). The changes in the VF in group 1 and group 2 were not statistically significant preoperatively and in the first month postoperatively (*P* > 0.05). In group 3, MD was −2.88 ± 1.20 (−3.63-2.10) dB, VFI was 97.45 ± 1.61 (89-92) dB, upper half VF sensitivity was 22.21 ± 1.54 (17-18.40) dB, and PSD was 2.19 ± 0.87 (1.2-2.02) dB at postoperative first month. The changes in group 3 were statistically significant preoperatively and in the first month postoperatively (*P *< 0.05).

**Conclusion:**

Dermatochalasis staging can predict the effect of dermatochalasis on the VF. This new dermatochalasis classification can be used in cases of equipment shortage. Visual field defects due to dermatochalasis can be corrected with blepharoplasty surgery.

**Main Points**

- It creates a visual field defect according to the stage of dermatochalasis.
- A new classification can be made according to the eyelash position in dermatochalasis, and the effect of blepharoplasty surgery can be evaluated in these stages.
- This new classification can be used in cases where VF testing cannot be performed, which is a legal issue.

## Introduction

Dermatochalasis is the laxity of the upper eyelid, causing visual field (VF) loss that makes it difficult to maintain primary gaze and read.^1^ Dermatochalasis staging was described by Jacobs in 2014 by examining many environmental and genetic factors^2^

However, there are many factors in this classification, and more practical classifications are needed. Upper eyelid blepharoplasty is a surgical procedure that eliminates the aesthetic appearance and the narrowing effect of dermatochalasis on the functional VF.^3^^,^^4^ Improvement in VF after blepharoplasty has been reported in the literature.^4^^,^^5^ Visual field testing is widely used during the evaluation of dermatochalasis. It is required to provide objective preoperative evidence of functional VF loss for medico-legal and insurance coverage criteria.^5^ In this study, a new and easier classification of dermatochalasis was made according to eyelash contact, mechanical effect, and eyelash inversion, and it was aimed to examine the preoperative and postoperative changes in VF during blepharoplasty according to this classification.

## Materials and Methods

**Figure 1.**
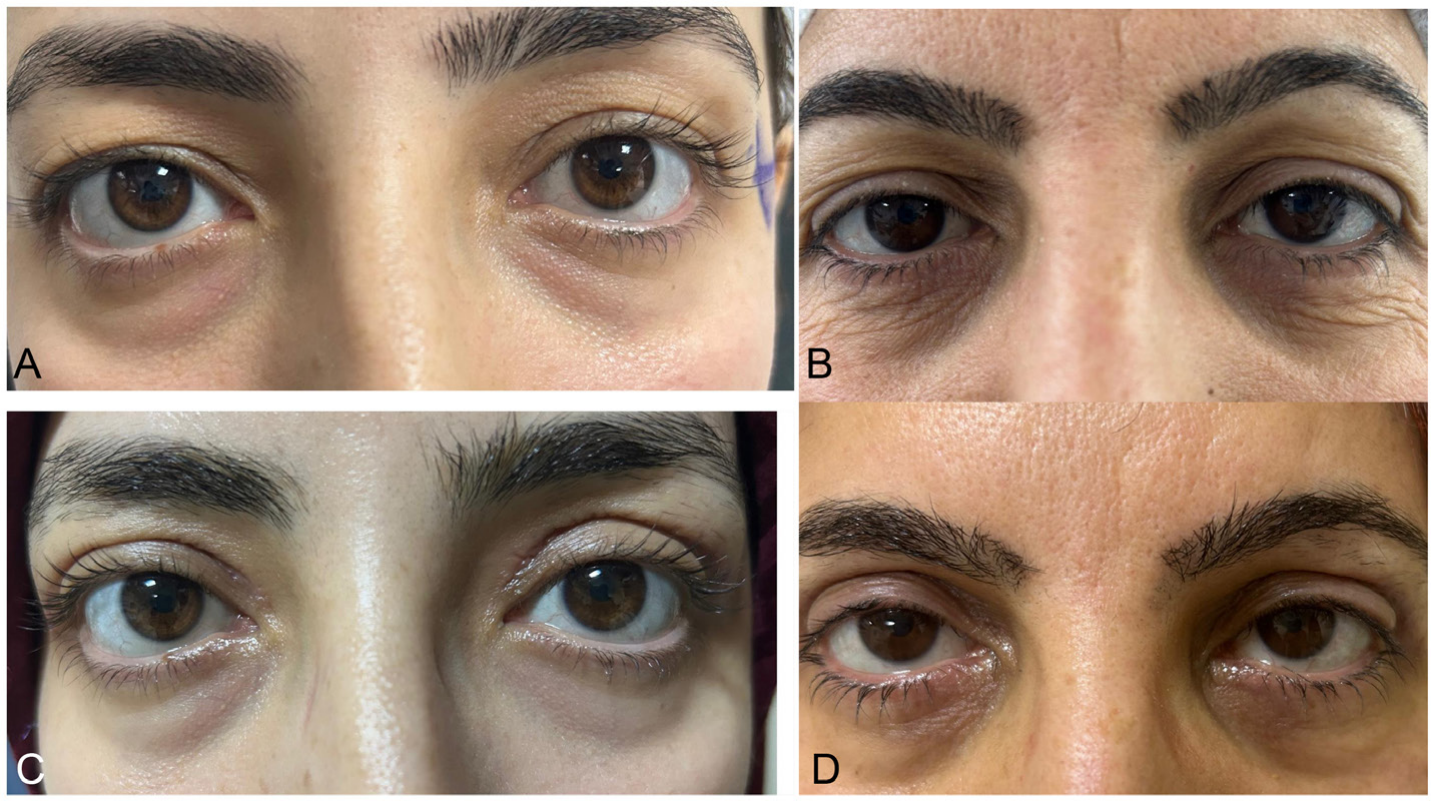
(A, B) Preoperative only dermatochalasis (stage 1), (C, D) postoperative first month view.

**Figure 2.**
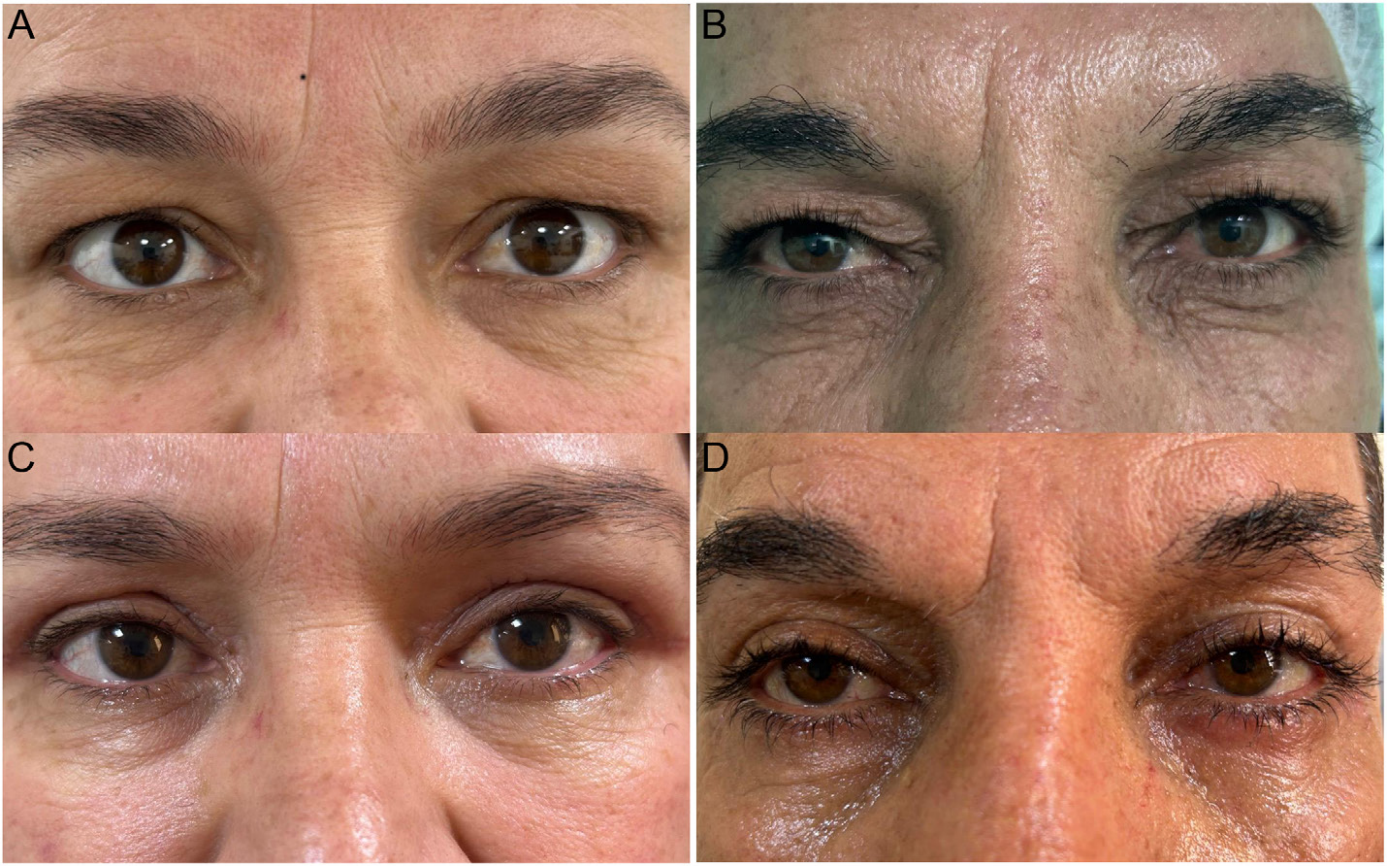
(A, B) Preoperative dermatochalasis with eyelashcontact (stage 2), (C, D)postoperative first month view.

**Figure 3.**
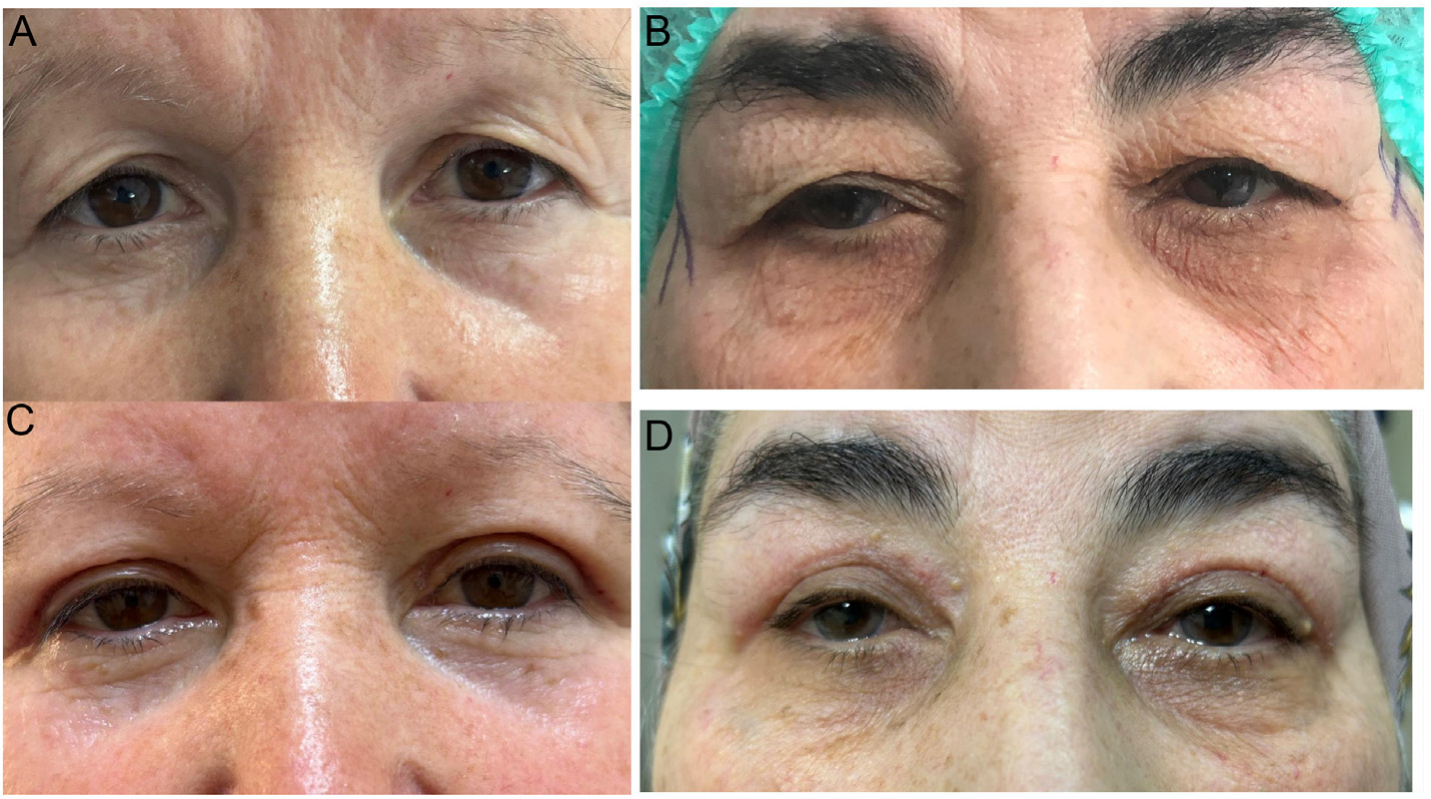
(A, B) Preoperative dermatochalasis causing eyelash inversion (stage 3), (C, D) postoperative first month view.

Our study prospectively evaluated cases who underwent upper eyelid blepharoplasty due to dermatochalasis in the author’s hospital eye clinic between February 2025 and April 2025. Informed written consent was obtained for the use of information and photographs of the cases. The study was approved by the ethics committee of Gazi Yaşargil Training and Research Hospital beforehand (Date: 07.02.2025 Approval no: 344). The Helsinki Declaration was followed throughout the study. Dermatochalasis classification of the cases was divided into 3 groups and 3 stages as upper eyelid not touching the eyelash (group 1) (stage 1) (Figure 1A and B), touching the eyelash but not inverting the eyelash (group 2) (stage 2) (Figure 2A and B), and touching the eyelash and inverting the eyelash (group 3) (stage 3) (Figure 3A and B). All staging was performed by the same ophthalmologist. All cases underwent a detailed ophthalmologic examination including best corrected visual acuity, anterior segment and fundus examination, and intraocular pressure measurement with a pneumotonometer, MRD1(margin reflex distance). Visual field test was performed preoperatively and at first month postoperatively using Humphrey 705 (Carl Zeiss Meditec Inc., Dublin, Calif, USA). The 30-2 algorithm was used to evaluate VF for all cases. In patients with appropriate fixation reliability criteria, mean deviation (MD), pattern standard deviation (PSD), and VF index (VFI) were calculated as the mean sensitivity of 38 points on the horizontal line (upper half VF sensitivity) in decibels (dB) where threshold scanning was performed on the upper VF. Patients with a history of eyelid or intraocular surgery, trauma, ptosis, MRD1 asymmetry greater than 1 mm, conjunctival or ocular surface problems, eyelid infection, dry eye syndrome, ocular surface disorder, any neuro-ophthalmologic disease, retinal disease, glaucoma suspicion that could affect the VF test, those who could not perform the VF test, and those who did not meet the reliability criteria of the VF test were not included in the study. Visual field test was performed with near vision correction according to age. All patients underwent blepharoplasty surgery with only skin excision under local anesthesia.

An a priori power analysis was conducted using the *G*Power 3.1* software to determine whether the sample size used in the study was statistically adequate. The analysis was based on a one-way ANOVA (fixed effects, omnibus) model, which was planned to be used under the assumption that the data met parametric test assumptions. The parameters used in the analysis were a medium effect size (Cohen’s *f* = 0.25), a significance level (α) of 0.05, and a total sample size of 60 participants (20 participants per group across 3 groups). According to these parameters, the calculated statistical power of the study was 1 – β = 0.87 (87%). This value exceeds the commonly accepted threshold of 80%, indicating that the sample size was sufficient to detect a medium-sized effect in the context of this study.

### Statistical Analysis

All statistical analyses were performed using IBM SPSS version 24 (IBM SPSS Corp.; Armonk, NY, USA). Descriptive statistics were expressed as mean ± SD, percentages, and minimum-maximum values for variables with a normal distribution. The normality of the data was assessed using the Shapiro–Wilk test. For quantitative comparisons, normally distributed variables were analyzed using the paired samples one-way ANOVA, while non-normally distributed variables were analyzed using the Wilcoxon signed-rank test. A *P*-value of < 0.05 was considered statistically significant.

## Results

A total of 60 cases were examined in this study, 20 in each group. The mean age of the cases was 42.7 ± 14.9 (28-75). There was a statistically significant difference between the groups, with the mean age being higher in group 3 (*P < 0.*05). Forty (62.5%) of the cases were female and 20 (37.5%) were male. There was no statistically significant difference between the groups in terms of gender (*P > 0.*05). Demographic characteristics according to groups are examined in Table 1. In group 1, MD was −3.47 ± 2.21 (−11.38-1.11) dB preoperatively, and −3.18 ± 1.41 (−5.63-2.10) dB at postoperative first month and this change was not statistically significant (*P > 0.*05). VFI was 95.18 ± 8.17 (59-91) preoperatively and 96.77 ± 1.79 (89-100) at postoperative first month and this change was not statistically significant (*P > 0.*05). The upper half VF sensitivity was 19.66 ± 5.67 (6.17-27.73) preoperatively and 20.23 ± 1.64 (19-29.91) at postoperative first month and this change was not statistically significant (*P > 0.*05). PSD was 3.91 ± 2.91 (1.51-10.09) preoperatively and 2.99 ± 0.97 (1.10-4.32) at postoperative first month and this change was not statistically significant (*P > 0.*05) (Table 2).

In group 2, MD was −4.01 ± 1.20 (−9.38-1.01) dB preoperatively and −3.78 ± 1.20 (−4.63-1.10) dB at postoperative first month and this change was not statistically significant (*P > 0.*05). VFI was 93.25 ± 7.17 (48–88) preoperatively and 94.55 ± 1.66 (88-98) at postoperative first month and this change was not statistically significant (*P > 0.*05). The upper half VF sensitivity was 19.55 ± 4.43 (6.17-19.13) preoperatively and 20.57 ± 1.64 (18-19.60) at postoperative first month and this change was not statistically significant (*P > 0.*05). PSD was 3.81 ± 2.88 (1.39-9.09) preoperatively and 3.37 ± 0.97(1.5-3.02) at postoperative first month and this change was not statistically significant (*P > 0.*05) (Table 3).

In group 3, MD was −5.92 ± 1.10 (−8.28-1.11) dB preoperatively and −2.88 ± 1.20 (−3.63-2.10) dB at postoperative first month and this change was statistically significant (*P < 0.*05). VFI was 90.25 ± 6.17 (48-88) preoperatively and 97.45 ± 1.61(89-92) at postoperative first month and this change was statistically significant (*P < 0.*05). The upper half VF sensitivity was 17.45 ± 4.43 (6.17-15.13) preoperatively and 22.21 ± 1.54 (17-18.40) at postoperative first month and this change was statistically significant (*P < 0.*05). PSDwas 5.01 ± 2.88 (1.39-9.09) preoperatively and 2.19 ± 0.87 (1.2-2.02) at postoperative first month and this change was statistically significant (*P < 0.*05) (Table 4).

## Discussion

In this study, it was observed that dermatochalasis narrows the upper VF when it touches the eyelash and inverts with mechanical effect, that is, when it is accepted as stage 3. As the skin becomes looser with age, dermatochalasis increases. It was observed that blepharoplasty surgery has an effect on the correction of this VF loss. With this newly implemented staging, VF loss becomes predictable and can be used as a legal protective factor in cases where there is a lack of equipment. Many healthcare institutions may not have a VF test; in this case, if dermatochalasis is stage 3, surgery can be performed by accepting the VF loss. Dermatochalasis is the loosening of the upper eyelid, which causes both functional and cosmetic problems. Difficulty in opening the eyelid, VF narrowing, and headache are some of the functional problems related to dermatochalasis.^6^ When planning surgery, creating a VF loss in order to perform the surgery under health insurance is a functional and legal problem in many countries.^7^ The American Academy of Ophthalmology (ASOPRS) published an ophthalmologic technology review in 2011 to evaluate preoperative indications and surgical outcomes for blepharoplasty and blepharoptosis repair.^8^ Several indicators were identified, including a total VF restriction of 12° or 24% of the superior VF, but almost all of the studies included in the review were Goldmann or automated VF tests. In fact, many insurers require a superior or lateral VF restriction of at least 12° or 30% of the superior VF to be covered by surgery.^9^ However, there is little literature that provides comparable or interchangeable data for these tests. Therefore, more practical evaluations are needed. VF and PSD are frequently used to evaluate VF loss. Many studies have reported that dermatochalasis causes a VF loss in the upper half due to mechanical effects.^10^^,^^11^ In this study, when dermatochalasis was staged, a VF loss developed in stage 3. There are studies reporting that the mechanical effect is eliminated and the VF loss is corrected because excess skin is removed after blepharoplasty surgery.^12^ In this study, it was observed that the VF loss of the cases with VF loss was corrected in the first month postoperatively. The staging of dermatochalasis was evaluated by Jacobs by examining many extrinsic and intrinsic factors.^2^ However, is it possible to predict the effect of dermatochalasis on VF by making a simpler staging since there are too many factors? Based on this question, this study examined the mechanical effect of dermatochalasis on eyelashes and performed the staging and showed that VF loss occurs when the eyelashes invert. Afterwards, when blepharoplasty was performed, the mechanical effect and pressure on the eyelashes decreased, so the eyelashes returned to their previous position and the postoperative VF loss was corrected. One of the strengths of this study is that all classifications were made by the same ophthalmologist, and its limitation is that it is an evaluation that cannot be measured numerically. This study is a newly defined dermatochalasis staging and is the first study to examine the effect of this staging on VF and the effect of blepharoplasty surgery on VF. As a result of this study, in restrictive cases where VF cannot be applied, it may be possible to legally perform blepharoplasty surgery by reporting a stage 3 dermatochalasis result. Fuller et al. reported that faster testing can be performed by using only the upper half field in VF testing in patients with dermatochalasis and ptosis and that margin reflex distance 1 (MRD1) correlates with VF results.^13^ Kim et al.^14^ who argued the opposite, reported that MRD1 was not affected after blepharoplasty and the VF was corrected. In the meta-analysis study reported by Rodrigues et al., it was reported that blepharoplasty surgery had no effect on VF and vision, but no classification was made.^15^ Labkovich et al.^16^ and Tsapakis et al.^17^ applied the median cut half field test (MCHT), an accelerated virtual reality (VR) suprathreshold half field perimetry algorithm, and reported that it correlates with VF loss. There are studies in the literature on cost-effective, fast, and easily applicable methods, but a full consensus has not been reached. In this study, the author think that this staging, which is performed according to the position of the eyelash affected by MRD1 in ptosis and does not require additional costly equipment, is easy to apply and has a low cost. As a result, dermatochalasis staging can make its effect on VF loss predictable. Visual field loss caused by dermatochalasis can be corrected with blepharoplasty surgery.

## Figures and Tables

**Table 1.** Demographic Characteristics of the Patients (P values in bold are statistically significant)

Variables	Group 1 (N:20)	Group 2 (N:20)	Group 3 (N:20)	*P*
Age (years)				
Mean ± SD	41.2 ± 4.3	42.3 ± 3.9	54.1 ± 4.0	**<0.05**
Range	28-75	30-68	40-75	
Gender [n(%)]				
Female	11 (13.75)	12 (15)	14 (17.5)	*> *0.05
Male	9 (11.25)	8 (10)	6 (7.5)	*> *0.05

*P* values in bold are statistically significant.

**Table 2.** Comparison of Values of Visual Field Indexes Preoperatively and Postoperative First Month (Group 1)

	Preoperative	Postoperative First Month	*P*
MD			
Mean ± SD (min-max)	−3.47 ± 2.21 (−11.38-1.11)	-3.18 ± 1.41 (−5.63-2.10)	*> *0.05^*^
VFI			
Mean ± SD (min-max)	95.18 ± 8.17 (59-91)	96.77 ± 1.79 (89-100)	*> *0.05^*^
The upper half visual field sensitivity			
Mean ± SD (min-max)	19.66 ± 5.67 (6.17-27.73)	20.23 ± 1.64 (19-29.91)	*> *0.05^*^
PSD			
Mean ± SD (min-max)	3.91 ± 2.91 (1.51-10.09)	2.99 ± 0.97 (1.10-4.32)	*> *0.05^*^

MD, mean deviation; PSD, pattern standard deviation; VFI, visual field index. *Statistically significant (*P* < 0.05).

**Table 3.** Comparison of Values of Visual Field Indexes Preoperatively and Postoperative First Month (Group 2)

	Preoperative	Postoperative First Month	*P*
MD			
Mean ± SD (min-max)	−4.01 ± 1.20 (−9.38-1.01)	-3.78 ± 1.20 (−4.63-1.10)	*> *0.05^*^
VFI			
Mean ± SD (min-max)	93.25 ± 7.17 (48-88)	94.55 ± 1.66 (88-98)	*> *0.05^*^
The upper half visual field sensitivity			
Mean ± SD (min-max)	19.55 ± 4.43 (6.17-19.13)	20.57 ± 1.64 (18-19.60)	*> *0.05^*^
PSD			
Mean ± SD (min-max)	3.81 ± 2.88 (1.39-9.09)	3.37 ± 0.97 (1.5-3.02)	*> *0.05^*^

MD, mean deviation; PSD, pattern standard deviation; VFI, visual field index. *Statistically significant (*P* < 0.05).

**Table 4.** Comparison of Values of Visual Field Indexes Preoperatively and Postoperative First Month (Group 3) (P values in bold are statistically significant)

	Preoperative	Postoperative First Month	*P*
MD			
Mean ± SD (min-max)	−5.92 ± 1.10 (−8.28-1.11)	−2.88 ± 1.20 (−3.63-2.10)	**0.05** ^*^
VFI			
Mean ± SD (min-max)	90.25 ± 6.17 (48-88)	97.45 ± 1.61 (89-92)	**0.05** ^*^
The upper half visual field sensitivity			
Mean ± SD (min-max)	17.45 ± 4.43 (6.17-15.13)	22.21 ± 1.54 (17-18.40)	**0.05** ^*^
PSD			
Mean ± SD (min-max)	5.01 ± 2.88 (1.39-9.09)	2.19 ± 0.87 (1.2-2.02)	**0.05** ^*^

MD, mean deviation; PSD, pattern standard deviation; VFI, visual field index. *Statistically significant (*P* < 0.05).

## Data Availability

The data that support the findings of this study are available on request from the corresponding author.
